# Penile strangulation with a plastic bottle neck: Intervened by an atypical instrument: A case report

**DOI:** 10.1016/j.ijscr.2021.106189

**Published:** 2021-07-08

**Authors:** Durga Neupane, Sudhir Kumar Singh, Awaj Kafle, Samir Chaudhary, Sushil Sharma Subedi, Sunit Chhetri

**Affiliations:** a B.P. Koirala Institute of Health Sciences, Dharan, Nepal; b Department of Surgery (Urology Division), B.P. Koirala Institute of Health Sciences, Dharan, Nepal; c Department of Surgery, B.P. Koirala Institute of Health Sciences, Dharan, Nepal

**Keywords:** Case report, Penile, Strangulation, Wire cutter, Plastic

## Abstract

**Introduction and importance:**

Strangulation of penis, a surgical emergency, is often encountered in patients with psychiatric disorders and patients attempting to increase sexual arousal. Materials ranging from metallic to non-metallic like plastic bottles are used by the patients. The important factors to be considered for the successful treatment include the availability of instruments and the surgical skills of the doctor.

**Case presentation:**

A 45 year-old man with a comorbidity of severe depression presented to Emergency with a two months long history of penile strangulation with a plastic bottle neck. The gross appearance of the penis showed edema and proliferative growths. He was intervened with a cable wire cutter as standard instrument failed to do so. The patient was discharged on the same day of intervention. However, he was lost to subsequent follow up.

**Discussion:**

Penile strangulation which is common in people with mental disorders should be considered as a surgical emergency as it can present with devastating complications. No specific methods and tools have been recommended for the removal of those objects. The shame felt by patient is the root cause for late surgical consultation and are prone to develop complications. Simple instruments can be used for the intervention provided good surgical skills are demonstrated.

**Conclusion:**

Common in psychiatric patients who deny medical attention due to shame, penile strangulation should be intervened quickly and simple instruments not routinely used in surgical practice can be effectively used to remove the offending objects.

## Highlights

• Penile strangulation is a surgical emergency.
• Offending objects can be metallic or non-metallic.
• Usually noted in psychiatric patients to augment sexual desire.
• Management is non-specific.
• A simple wire cutter can be used to cut the plastic bottle neck.

## 1 Introduction

Entrapment or strangulation of the penis is a rare emergency situation that necessitates urgent treatment for decompression of the penis. Strangulation objects can be either metallic [1] or non-metallic [2] and are usually associated with an attempt to maintain a longer erection. It requires urgent intervention and treatment as it may affect vascular injury or even necrosis [3]. No standard of care has been found to be greater, with each case managed individually according to its clinical findings and operative settings [4].

Here we present a case of penile strangulation in a patient with severe depression. This case has been reported in line with SCARE criteria [5].

As the patient was a case of severe depression, written informed consent was obtained from his brother for the surgical procedure and to report this case along with use of images for publication.

## 2 Case presentation

**Fig. 1:**
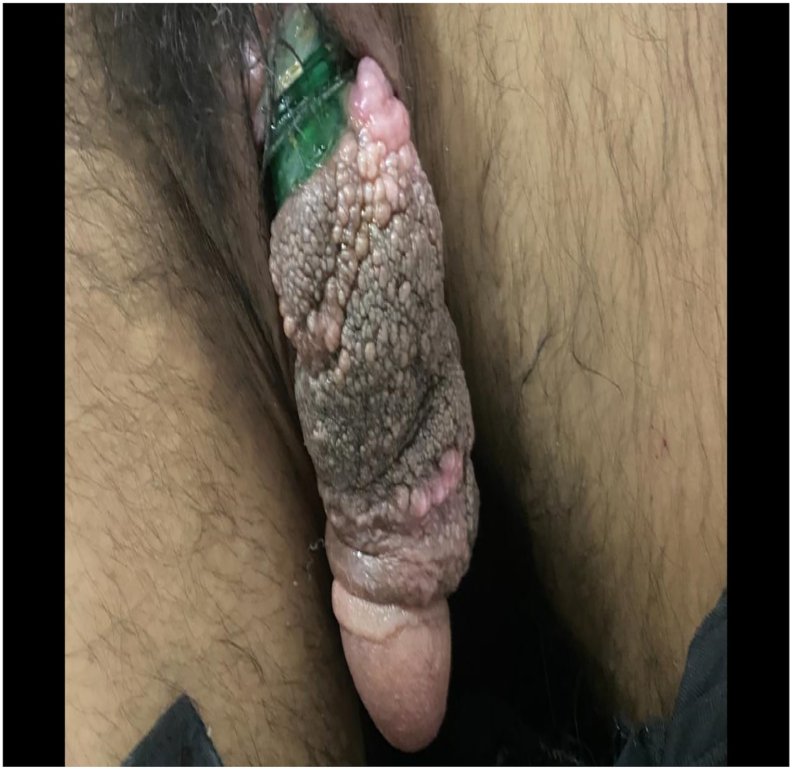
Penile strangulation by a plastic bottle neck with associated edema and proliferative growth.

**Fig. 2:**
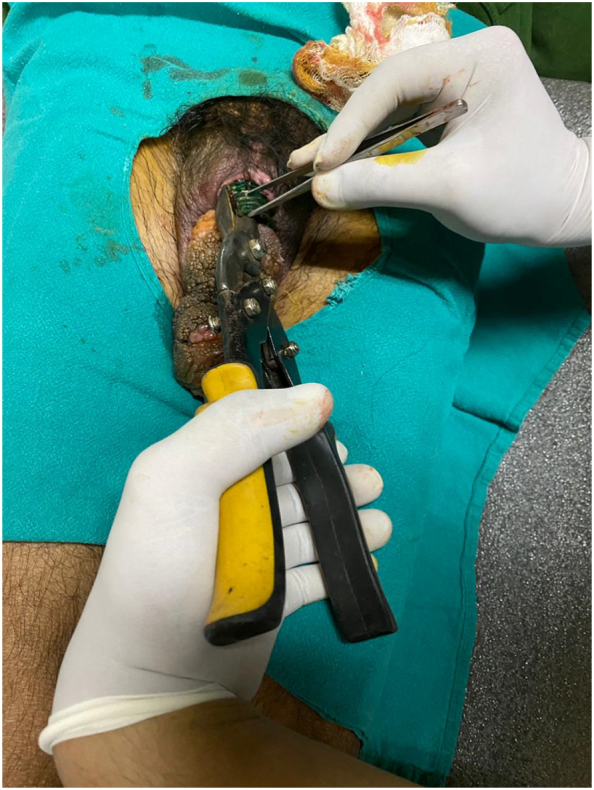
This instrument was unable to cut the bottle neck.

**Fig. 3:**
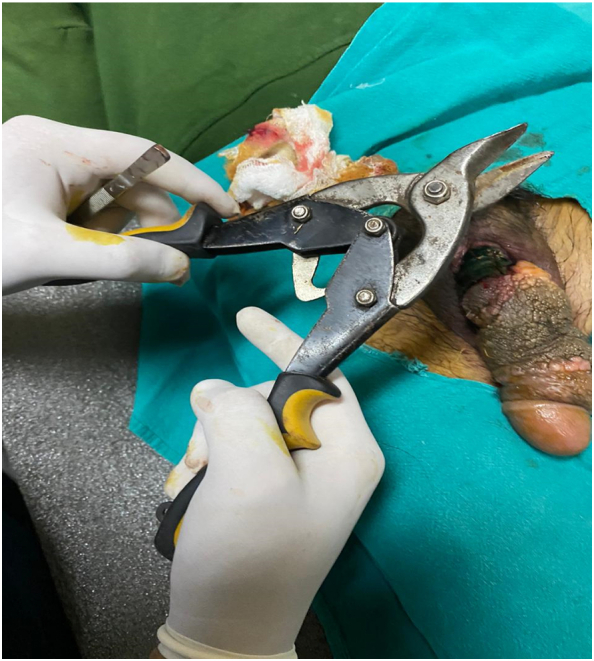
The bottle neck was successfully cut with this simple cable wire cutter which is not routinely used in surgical practice.

A 45 year-old man came to Emergency with penile strangulation by a plastic bottle neck for 2 months ago as shown in Fig. 1. The patient had prior diagnosis of severe depression with multiple suicidal attempts. Since he was mentally ill, he did not reveal the incident with his family members. In addition to it, the reason for strangulation was not deduced as we could not elicit the medical history properly. The shaft of penis, which was distal to the bottle neck, was oedematous, congested with proliferative growths. The offending foreign body was intervened with standard equipment as shown in Fig. 2 but was failed. Then, a simple cable wire cutter as shown in Fig. 3 could cut the bottle neck and the patient was discharged with symptomatic treatment. The patient was lost to follow up.

## 3 Discussion

Penile strangulation is an unusual clinical condition with the first case being reported in 1755 by Gauthier [6]. Since then, nearly 60 cases have been reported in the world literature [3]. It could be acute or chronic lasting for months.

Strangulation of the penis is often found in psychiatric patients who pursued to improve their sexual performance by prolonging the duration of erection or as part of autoerotic games. The patient can use several objects, ranging from non-metallic objects (adhesive tape, piping, hair, plastic bottle etc.), to metallic (ring, keyring, etc.). Two schizophrenic patients have been reported to have penile strangulation and amputation [7]. Our patient had a primary diagnosis of severe depression with multiple suicidal attempts.

The choice of method for elimination depends upon type, incarceration time, size, trauma grade, and availability of the equipment [8], [9], [10]. Our patient was managed with a cable wire cutter since the standard instrument failed to do so. So, treatment can be done according to the available settings and also depends on the operative skills and techniques.

## 4 Conclusion

Penile strangulation is common in psychiatric patients and warrants emergency management to preserve the organ function. Each case is managed exclusively according to its clinical findings and operative settings. Management depends on the type and size of the constricting object, available instruments, time after incarceration, degree of injury and experience of the surgeons. Different methods and tools can be used depending upon clinical scenarios.

## Ethical approval

Not required.

## Funding

None.

## Guarantor

Durga Neupane.

## Registration of research studies

Not applicable.

## Consent for publication

As patient was a known case of severe depression, written informed consent was obtained from the patient's brother for publication of this case report and accompanying images. A copy of the written consent is available for review by the Editor-in-Chief of this journal on request.

## Provenance and peer review

Not commissioned, externally peer-reviewed.

## CRediT authorship contribution statement

Durga Neupane(DN), Awaj Kafle(AK), Sushil Sharma Subedi(SSS), Sudhir Kumar Singh(SKS) = Study concept, Data collection, and surgical therapy for the patient.

Durga Neupane(DN), Awaj Kafle(AK), Sudhir Kumar Singh(SKS), Samir Chaudhary(SC) = Writing- original draft preparation.

Durga Neupane(DN), Awaj Kafle(AK), Sunit Chhetri(SC) = Editing and writing.

Durga Neupane(DN), Awaj Kafle(AK), Sudhir Kumar Singh(SKS) = senior author and manuscript reviewer.

All the authors read and approved the final manuscript.

## Declaration of competing interest

None.
